# Weight Loss Therapies and Hypertension Benefits

**DOI:** 10.3390/biomedicines12102293

**Published:** 2024-10-10

**Authors:** Vasiliki Katsi, Eleni Manta, Christos Fragoulis, Konstantinos Tsioufis

**Affiliations:** First Department of Cardiology, Hippokration General Hospital, National and Kapodistrian University of Athens Medical School, 114 Vasilissis Sofias Avenue, 11527 Athens, Greecechristosfragoulis@yahoo.com (C.F.);

**Keywords:** obesity, weight loss therapies, blood pressure, hypertension, phentermine/topiramate, orlistat, naltrexone/bupropion, GLP-1 receptor agonists, tirzepatide, bariatric surgery

## Abstract

Obesity and hypertension have become an international health issue, with detrimental consequences on patients. Obesity and hypertension share common pathophysiological mechanisms, such as overactivity of the renin–angiotensin–aldosterone and the sympathetic nervous systems, insulin resistance, and disruption of the leptin pathway. Approved therapies for obesity and overweight include phentermine/topiramate, orlistat, naltrexone/bupropion, the glucagon-like peptide-1 receptor agonists liraglutide and semaglutide, tirzepatide, and bariatric surgery. This review gives the clinical data in a thorough manner and explains in detail how each of the previously mentioned therapies affects blood pressure levels.

## 1. Introduction

Obesity is defined as the increase in the amount of fat that poses a health concern [1]. The body mass index (ΒΜΙ) is used for the quantification of body fat, and the somatometric characteristics of the person are taken into account for its calculation. An individual with a BMI over 25 kg/m^2^ is classified as overweight, while a BMI over 30 kg/m^2^ indicates that a person is obese [2]. Obesity is associated with the occurrence of diseases that serve as cardiovascular risk factors such as type II diabetes mellitus and hyperlipidemia. On top of that, obesity is recognized as an independent risk factor for cardiovascular morbidity and mortality [3]. The prevalence of obesity and overweight in both adults and children is on the rise. The percentage of obese adults increased from 7% to 16% between 1990 and 2022, and at the same time the percentage of obese children and adolescents increased from 2% to 8% worldwide [1].

Arterial hypertension is one of the most significant health issues globally, as it affects 35–40% of adults and is predicted to impact more than 1.5 billion people by 2025 [4]. Hypertension is the greatest avoidable risk factor for cardiovascular disease, as it contributes to 54% of strokes, 30% of end-stage renal disease, and 47% of coronary heart disease, accounting for 0.4 million deaths (or 14.5% of all deaths) and 6.3 million years of disability [5]. Almost half of patients with hypertension are not aware that they suffer from this disease, less than half of those patients do not receive treatment, while only 20% of patients have their blood pressure regulated [6].

This article aims to present the correlation between these two conditions and then to analyze the effect of interventions used to treat obesity on blood pressure levels.

## 2. The Link between Obesity and Hypertension

There is substantial evidence linking these two health conditions, with obesity being a key risk factor for the development of hypertension. Research indicates that obese individuals have a 3.5 times higher risk of developing hypertension [7]. In fact, in all age groups, the prevalence of hypertension in obese individuals can vary from 60% to 77%, while in people of normal weight this prevalence is around 34% [8].

**Figure 1:**
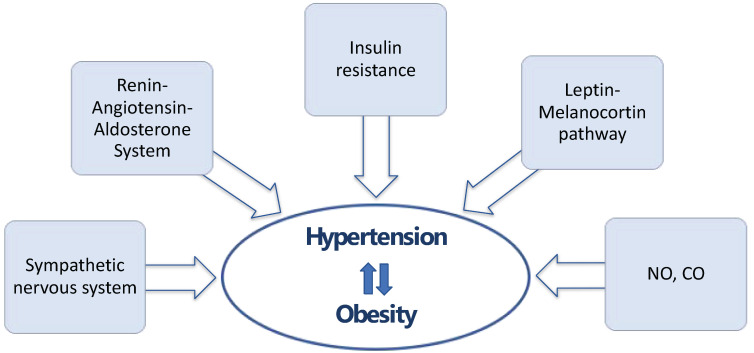
Pathophysiological mechanisms that contribute to the development of arterial hypertension and obesity.

The fact that arterial hypertension and obesity often coexist is grounded on several causes. First, these two pathological entities are affected by the same environmental factors, such as smoking, increased alcohol and salt intake, and limited physical activity [7]. In addition, overlapping pathophysiological mechanisms have emerged, which are involved in the development of both conditions (Figure 1).

### 2.1. Sympathetic Nervous System

A key common pathophysiological mechanism of arterial hypertension and obesity is the overactivation of the sympathetic nervous system (SNS). Arterial hypertension is characterized by sympathetic overstimulation, and this activation is initiated by the central nervous system. Further, peripheral factors contribute to chronically elevated blood pressure, such as downregulation of peripheral α1-adrenergic receptors, impaired norepinephrine reuptake from sympathetic nerve endings, impaired reflexes of baroreceptors in the cardiopulmonary region which limit the adrenergic outflow, impaired arterial chemoreceptors, as well as interaction with the renin–angiotensin–aldosterone system [9].

The SNS is also stimulated by obesity. Muscle and renal SNS activity seems to rise with even small weight gains and this rise is greater in people who are both obese and hypertensive [10]. Abnormal adipokine production from adipose tissue, baroreceptor dysfunction, interaction with the renin–angiotensin–aldosterone system, and insulin resistance are among the causative pathways of SNS activation in obesity [10]. The simultaneous occurrence of obesity and hypertension is associated with a higher level of sympathetic activity compared with the sympathetic activity observed in relation to each condition separately. Furthermore, studies have demonstrated that individuals with central fat have higher blood pressure values and stronger sympathetic activation, making the location of fat particularly significant [7].

### 2.2. Renin–Angiotensin–Aldosterone System

Sympathetic activation in hypertension also depends on the interaction with the renin–angiotensin–aldosterone system, as mentioned above. Circulating angiotensin stimulates the SNS by not only its effect on the brain, but also through its effect on sympathetic nerve endings that facilitate neurotransmission [11]. In addition, renal sympathetic activation facilitates renin release from the paraglomerular apparatus, leading to increased aldosterone levels, sodium and water retention, renal vasoconstriction, and microalbuminuria [12].

Numerous studies showcase that the renin–angiotensin–aldosterone system is also activated in obesity, mainly because of the interaction with the SNS, which leads to the release of renin from the kidneys [10]. Accumulation of fat around the kidney also plays a role, leading to compression of the kidney, which results in renin secretion [10]. Additionally, adipocytes seem to have their own intrinsic renin–angiotensin–aldosterone system that produces angiotensinogen and angiotensin II [10].

### 2.3. Insulin Resistance

The adrenergic overstimulation that occurs in hypertension also has a humoral origin, since it is triggered by metabolic alterations such as the hyperinsulinemic state and subsequent insulin resistance, as the stimulating effect of insulin has already been documented [13]. In fact, hyperinsulinemia and subsequent insulin resistance are the main mechanisms leading to sympathetic activation in obesity [9]. It is important to emphasize that, in this as well as in other situations, the SNS often regresses the stimulation it receives, thus forming a vicious circle in which the components involved reinforce each other. In the case of insulin, resistance to it is increased by sympathetic effects on reduced vasoconstriction in skeletal muscle regions, which leads to a further increase in insulin secretion and therefore further sympathetic activation [14]. In addition, insulin promotes sodium reabsorption by the kidneys, while it also appears to affect vasodilation through its effects on the vascular endothelium [7].

### 2.4. Leptin–Melanocortin Pathway

Another mechanism involved in the occurrence of hypertension and obesity is leptin and the leptin–melanocortin pathway. Leptin is a cytokine that has a role in energy homeostasis, as it negatively affects brain regions related to energy intake by informing the brain about the amount of fat stored in the body. These actions are driven by its binding to melanocortin receptors in the hypothalamus and in sympathetic neurons of the spinal cord [15]. Leptin levels appear elevated in the plasma of hypertensive patients, paralleling the magnitude of adrenergic hyperfunction [9]. In addition, leptin levels are paradoxically elevated in obese individuals, so it has been hypothesized that a state of leptin resistance occurs, suppressing its appetite-modulating effect but not its SNS-stimulating effect [10].

### 2.5. New Mediators in Hypertension

**Figure 2:**
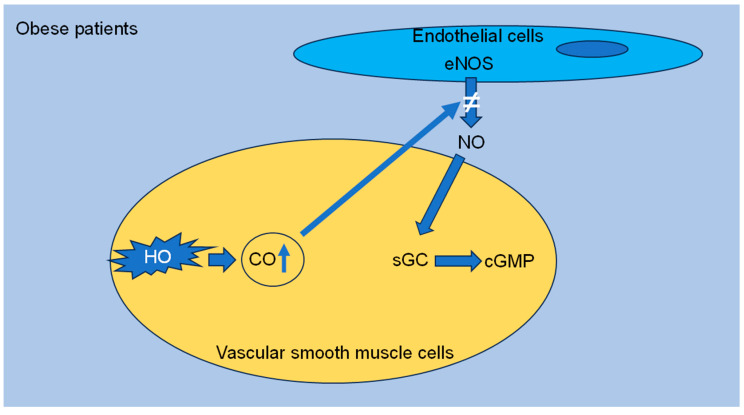
Proposed mechanism of the role of carbon monoxide in obese patients. In obese patients, a hypothesis has developed that HO-derived CO production is increased, resulting in inhibition of eNOS and decreased concentration of NO. This ends up in downregulation of cGMP, vasoconstriction, and endothelial dysfunction, eventually promoting hypertension. cGMP: guanosine 3′,5′-cyclic monophosphate, CO: carbon monoxide, eNOS: endothelial nitric oxide synthase, HO: heme oxygenase, NO: nitric oxide, sGC: soluble guanylyl cyclase.

Nitric oxide (NO) is a signaling molecule that is produced naturally by endothelial cells through the activation of endothelial nitric oxide synthase (eNOS), stimulating the increase in intracellular guanosine 3′,5′-cyclic monophosphate (cGMP) levels in vascular smooth muscle cells (VSMCs) to induce vasodilation. Studies conducted over the last few decades have shown that the reduction in NO production, leading to vasoconstriction and endothelial dysfunction, represents the earliest stages in the development of hypertension [16]. Furthermore, carbon monoxide (CO) has been found to relax blood vessels in various organs. The primary source of CO in the vascular system is heme oxygenase (HO)-2, which is present in both endothelial and vascular smooth muscle cells. Similar to NO, CO’s ability to dilate blood vessels occurs through the activation of soluble guanylyl cyclase (sGC), elevation of cGMP, and stimulation of high-conductance Ca^2+^-activated K^+^ channels. The triggering of K^+^ channels results in membrane hyperpolarization, which hinders calcium entry from voltage-activated Ca^2+^ channels. Under typical circumstances, CO is generated from the degradation of intracellular heme by heme oxygenase. Subsequently, CO triggers sGC to elevate intracellular cGMP levels, thereby enhancing the activity of K^+^ channels to induce vasodilation. CO might also directly influence K^+^ channels to boost their activity. The relationship between vascular NO and CO is highly complex. Both gases can activate sGC to elevate cGMP levels and induce vasodilation. Previous research has demonstrated that low levels of CO (0.001 to 0.1 μmol/L) can prompt NO release, while higher levels of CO (≥1 μmol/L) impede eNOS. In instances where CO production is significantly heightened in VSMCs, CO can impede eNOS in endothelial cells, reducing NO production and diminishing NO-mediated increases in cGMP, leading to decreased VSMC relaxation and causing vascular dysfunction [17]. As presented in the obese Zucker rat model of the metabolic syndrome, HO-derived CO production is increased and promotes hypertension and arteriolar endothelial dysfunction with the mechanisms described above. These results could explain another mechanism that contributes to hypertension in obese patients [18] (Figure 2).

## 3. Blood Pressure and Weight Loss

Data from epidemiological studies show that the reduction in body weight leads to a reduction in blood pressure levels. In fact, the relationship between these two variables is linear, as it has been shown that a 1 kg reduction in body weight through lifestyle interventions can lead to a 1 mmHg reduction in systolic blood pressure levels [19,20].

The underlying pathophysiological mechanisms for this association are likely based on adipose tissue. A reduction in adipose tissue appears to reduce induced inflammation, thereby limiting inflammation-induced hardening of the arteries, as well as improving insulin resistance [8]. In addition, the reduction in perirenal fat leads to the reduction in SNS stimulation and the limitation of sodium retention, as these occur in a manner described above [8]. According to the most relevant recent Clinical Consensus of the European Society of Cardiology, it is recommended to maintain a stable BMI within normal limits (20–25 kg/m^2^) to reduce both blood pressure levels and cardiovascular risk. In addition, antihypertensive treatment is recommended in patients with obesity and confirmed arterial hypertension (office blood pressure values ≥ 140/90 mmHg) or in patients with blood pressure values of 130–139/80–89 mmHg and high cardiovascular risk. Furthermore, the importance of healthy dietary interventions is emphasized as a first step in the treatment of obesity and hypertension, before the initiation of antihypertensive treatment [21].

## 4. Lifestyle Interventions

Interventions for lifestyle involve dietary adjustments, physical activity, and psychological support. In dietary interventions, the goal is usually to create an energy deficit of 500–750 kcal/day. It is important to customize these interventions based on individual body weight and activity levels. Weight reduction of 5–10% can be attained through different nutritional and multidisciplinary methods, but sustaining these effects is crucial [21]. The recommendation is to target a stable and healthy BMI (e.g., 20–25 kg/m^2^) and waist circumference values (e.g., <94 cm in men and <80 cm in women) to lower the risk of high blood pressure and cardiovascular disease. Embracing a healthy and well-balanced diet, such as the scientifically supported Mediterranean or Dietary Approaches to Stop Hypertension (DASH) diet, is advised [22]. Physical activity interventions generally have modest impacts on weight loss but are essential for maintaining weight loss and decreasing overall cardiovascular risk [21]. It is recommended to engage in moderate-intensity aerobic exercise for ≥150 min/week (≥30 min, 5–7 days/week) or alternatively 75 min of vigorous-intensity aerobic exercise per week over 3 days. This should be supplemented with low- or moderate-intensity dynamic or isometric resistance training (2–3 times/week) to lower the risk of high blood pressure and cardiovascular disease [22]. Lastly, psychological interventions encompass stress and depression management, as well as the adoption and maintenance of the aforementioned healthy behaviors [21]. The average reduction in systolic blood pressure (SBP) due to regular aerobic exercise is 5 mmHg, and the average reduction in diastolic blood pressure (DBP) is 4 mmHg. Adequate reduction in salt (sodium) in the diet results in a similar average reduction in blood pressure. In addition, moderate alcohol consumption can lead to a decrease in SBP by around 4 mmHg and DBP by 2.5 mmHg. For individuals who are overweight or obese, losing 3% to 9% of their weight results in approximately 3 mmHg reductions in both systolic and diastolic blood pressure. Following the DASH diet, which is low in saturated fats and high in fruits and vegetables, can lead to an average reduction of 11 mmHg in SBP and 5.5 mmHg in DBP [23].

## 5. Pharmacological Therapy for Weight Reduction

**Figure 3:**
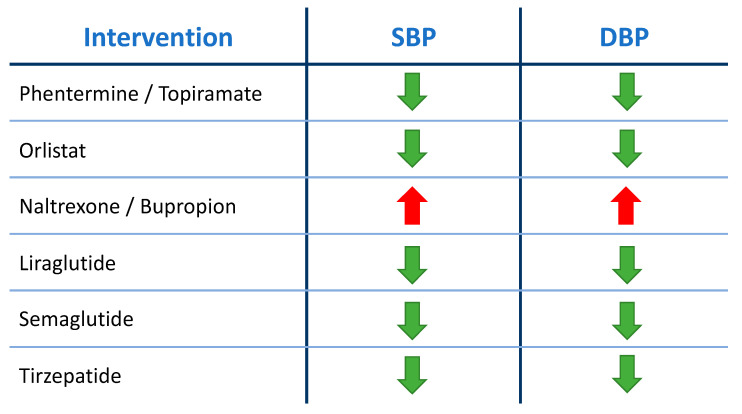
Pharmacological therapies for weight reduction and their effect on blood pressure levels.

The increasing morbidity linked to obesity has prompted the creation of medications designed to effectively treat the condition. The medicinal substances approved by the US Food and Drug Administration (FDA) for chronic weight management are the following: phentermine/topiramate, orlistat, naltrexone/bupropion, the glucagon-like peptide-1 receptor agonists liraglutide and semaglutide, and tirzepatide. The same drugs have been approved by the European Medicines Agency (EMA) for the treatment of obesity in the European Union, except for phentermine/topiramate. The mechanisms by which these medicinal substances lead to a reduction in body weight are analyzed below. Also, the available evidence is analyzed regarding their effects on blood pressure levels, and is schematically illustrated in Figure 3.

### 5.1. Phentermine/Topiramate

#### 5.1.1. Mechanism of Action

Phentermine is a sympathomimetic amine that suppresses appetite by promoting the release of norepinephrine. Topiramate upgrades the action of gamma-aminobutyric acid (GABA) and inhibits carbonic anhydrases, which are excitatory neurotransmitters. Topiramate was initially approved for the treatment of epilepsy, while the mechanisms through which it induces weight loss have not been completely illustrated [24]. The fixed combination of these two substances was created in order to enhance the benefits of both, while concurrently lowering the negative effects that each of the chemicals caused [24]. The FDA has authorized this combination in one formulation for the treatment of obesity; however, the EMA has rejected its use in the European Union due to safety concerns.

#### 5.1.2. Clinical Evidence on Blood Pressure

Several phase III clinical trials examined the safety and effectiveness of phentermine/topiramate in obese participants. In the EQUIP trial, 1267 people with BMI ≥ 35 kg/m^2^, with or without hypertension, were treated with phentermine/topiramate in low dose (phentermine 3.75 mg/topiramate 23 mg per day) or full dose (phentermine 15 mg/topiramate 92 mg per day) or a placebo for 56 weeks. Individuals who were administered the medication experienced a substantial decrease in both SBP and DBP after 56 weeks of treatment [25]. A similar finding was demonstrated in the CONQUER trial, which concluded that phentermine/topiramate substantially lowered blood pressure in patients who were overweight or obese and had two or more comorbidities. The correlation between weight loss and cardiovascular outcomes was highlighted by the fact that participants who achieved a 10–15% decrease in their baseline weight saw the greatest reduction in blood pressure [26]. In the EQUATE trial, 600 subjects with BMI 30–45 kg/m^2^ with or without arterial hypertension were randomized to topiramate 46 mg, topiramate 92 mg, phentermine 7.5 mg, phentermine 15 mg, phentermine/topiramate 7.5/46 mg, phentermine/topiramate 15/92 mg, or a placebo. Subjects who received phentermine/topiramate showed higher reductions in SBP than those who received a placebo, while DBP differences were not statistically significant between the examined groups [27]. The SEQUEL trial was an extension of the CONQUER study for another 52 weeks, where 676 participants of the CONQUER trial continued the assigned drug from the initial randomization. Of the trials mentioned above, this was the only one in which the difference in the reduction in blood pressure between the drug-treated group and the placebo group was not statistically significant [28].

#### 5.1.3. Mechanisms Affecting Blood Pressure

The apparent underlying mechanism by which phentermine/topiramate lowers blood pressure is weight reduction. Additional mechanisms that have been suggested include diuresis induced by carbonic anhydrase inhibition or neurotransmitter inhibition, as mentioned above [24].

### 5.2. Orlistat

#### 5.2.1. Mechanism of Action

Orlistat is a long-acting gastrointestinal lipase inhibitor. It acts in the lumen of the stomach and the small intestine where it forms a covalent bond with the active serine center of gastric and pancreatic lipases. Thus, the deactivated enzyme is unable to hydrolyze the dietary fat, resulting in a reduction in its absorption by ~30% [29]. Orlistat usage has been linked to comparatively mild weight reduction, and it causes gastrointestinal disturbances that usually lead to its discontinuation by a significant proportion of patients [30].

#### 5.2.2. Clinical Evidence on Blood Pressure 

Orlistat’s effectiveness in lowering blood pressure has been assessed in a number of clinical trials and meta-analyses. Sahebkar et al. conducted a meta-analysis of 27 randomized controlled trials, with a total of 8150 individuals, in order to compare the effects of orlistat against placebo on blood pressure. Patients who were overweight or obese and had concomitant conditions including type II diabetes mellitus and hypertension were included in the selected trials. In this meta-analysis, orlistat was found to have a significant effect on lowering SBP by 1.15 mmHg and DBP by 1.07 mmHg, with no significant difference found between subgroups based on dosage (360 mg vs. 180 mg daily) and length of treatment (more vs. less than 12 months) [31]. These findings suggest that orlistat’s ability to lower blood pressure is consistent across a range of patient demographics and medication durations.

When paired with dietary changes, orlistat has been demonstrated to dramatically improve blood pressure management in obese people with hypertension. For instance, a meta-analysis conducted in 2002 by Sharma and Golay found that obese individuals with uncontrolled diastolic hypertension or isolated systolic hypertension who were treated with orlistat had higher reduction in blood pressure, both systolic and diastolic, than patients who were given a placebo. In particular, the orlistat group saw a reduction in SBP by 9.4 mmHg and in DBP by 7.7 mmHg. An interesting finding of this particular study is that the greatest drop in blood pressure was observed in patients who lost more than 10% of their body weight [32].

#### 5.2.3. Mechanisms Affecting Blood Pressure

The blood pressure benefit of orlistat has been tested and documented in obese individuals with or without comorbidities such as hypertension or diabetes mellitus. The main mechanism through which orlistat reduces blood pressure is the loss of body weight, which, as previously mentioned, lessens SNS overstimulation. Orlistat also lowers blood pressure in part because of its effects on endothelial function and oxidative stress. Research has indicated that orlistat improves the function of microvascular endothelial cells, potentially by reducing triglyceride and LDL levels, which is essential for preserving normal blood pressure levels [33].

### 5.3. Naltrexone/Bupropion

#### 5.3.1. Mechanism of Action

Naltrexone is an antagonist of opioid receptors. It mainly inhibits the actions of endogenous opioids, which are recognized for their ability to regulate cardiovascular, reward, and pain responses. Naltrexone may have an impact on food intake and body weight by acting on the melanocortin system of the hypothalamus, which includes opiate neurons [34]. Bupropion is a weak nicotinic acetylcholine receptor antagonist, which also affects the reuptake of catecholamines, specifically dopamine and norepinephrine. Bupropion administration facilitates changes in the concentrations of dopamine and norepinephrine in the brain and may also modify the activity of the neurons that emit these chemicals by preventing their clearance. The melanocortin system is influenced by dopamine and norepinephrine, and a number of characteristics of obesity are associated with a reduction in the hypothalamic dopaminergic tone [34]. The purpose of combining these two ingredients was to maximize their weight reduction benefits through their synergy.

#### 5.3.2. Clinical Evidence on Blood Pressure 

The efficacy of this combination in weight regulation was evaluated by four Phase III clinical trials. The CONTRAVE Obesity Research I (COR-I) trial included 1742 patients with a BMI of 30–45 kg/m^2^ or a BMI of 27–45 kg/m^2^ who also had dyslipidemia or arterial hypertension. The trial compared the effectiveness of naltrexone/bupropion at dosages of 16/360 mg or 32/360 mg to a placebo in treating obesity. The combination therapy caused the mean SBP and DBP to slightly and temporarily rise from baseline [35]. The CONTRAVE Obesity Research II (COR-II) trial examined the effects of the combination naltrexone/bupropion 32/360 mg vs. a placebo in ~1500 individuals with a BMI of 30–45 kg/m^2^ or a BMI of 27–45 kg/m^2^ who also had dyslipidemia and/or arterial hypertension. Despite the substantial drop in body weight shown in the medication-treated group, there was no statistically significant difference from the placebo group in the patients’ blood pressure values during the follow-up period [36]. The COR Behavior Modification (COR-BMOD) trial included 793 overweight or obese individuals who were already following a balanced diet and physical activity program. Both treatment groups reported a decrease in SBP and DBP, with the placebo group experiencing a greater reduction than the group receiving naltrexone/bupropion 32/360 mg per day [37]. The effectiveness of the combination of naltrexone and bupropion in weight loss has also been demonstrated in overweight or obese patients with type II diabetes mellitus. When the combination was administered to 500 individuals who met these criteria, Hollander and colleagues saw a substantial reduction in body weight when compared with a placebo. The combination in this trial reduced the levels of SBP and DBP, with no statistically significant difference between the individuals who received the medication and the placebo group [38].

The above clinical studies had equivocal results regarding the effect of naltrexone/bupropion on blood pressure levels. The most recent relevant meta-analysis attempted to clarify the picture. Jiang et al. observed that the combination treatment caused a slight rise in both SBP and DBP. More specifically, a small but statistically significant increase in SBP by 1.34 mmHg and DBP by 0.93 mmHg was observed [39].

#### 5.3.3. Mechanisms Affecting Blood Pressure

The chemical processes by which naltrexone and bupropion regulate blood pressure involve interactions with the central nervous system that affect inflammatory processes and neurotransmission pathways. Naltrexone is an opioid receptor antagonist. Thus, it reduces the effects of endogenous opioids. These substances act on receptors in the endothelium of the vessels and in the heart, and cause vasodilation with consequent hypotensive effects. Therefore, naltrexone administration may result in an increase in blood pressure through inhibition of the intrinsic actions of endogenous opioids [40]. Bupropion mainly operates by preventing dopamine and norepinephrine from being reabsorbed, which raises their levels. Because norepinephrine increases SNS activity, blood pressure may also rise as a result. Moreover, bupropion’s actions on the neurological system and potentially blood pressure control are attributed to its noncompetitive antagonistic activity at nicotinic acetylcholine receptors [41]. The hypothalamus melanocortin system, which is important for energy balance and cardiovascular control, is also impacted by naltrexone/bupropion combination treatment [34]. The regulation of this route by naltrexone and bupropion may be responsible for the reported cardiovascular effects, as this system’s activation can affect blood pressure in the way mentioned above.

### 5.4. Glucagon-like Peptide-1 Receptor Agonists

#### 5.4.1. Mechanism of Action

Glucagon-like peptide-1 (GLP-1) and gastric inhibitory peptide-1 (GIP) are intestine hormones (also called ‘incretins’) that are secreted in reaction to stimuli from digested nutrients and have the ability to glucose-dependently trigger the release of insulin from pancreatic cells [42]. The natural GLP-1 hormone has a relatively limited half-life because of its rapid breakdown by the enzyme dipeptidyl peptidase-4 (DPP-4). In order to achieve a longer operation and more enduring therapeutic advantages, GLP-1 receptor agonists were specifically designed to be resistant to degradation by DPP-4 [43].

GLP-1 receptor agonists were initially created to treat type II diabetes mellitus, by amplifying the metabolic effect of the gut hormone GLP-1. GLP-1 delays stomach emptying, increases postprandial insulin secretion, inhibits glucagon release, and suppresses appetite [44]. However, they were also proven to be useful in lowering body weight. Thus, once-daily subcutaneous injection of liraglutide 3.0 mg and once-weekly subcutaneous administration of semaglutide 2.4 mg were studied and approved as anti-obesity medications. GLP-1 receptor agonists lower body weight in a variety of ways, notably by enhancing satiety and reducing appetite, which in turn leads to less calorie consumption [45].

#### 5.4.2. Clinical Evidence on Blood Pressure

##### Liraglutide

Liraglutide has been licensed as a weight-loss medication for obese individuals with or without type II diabetes mellitus, following phase III clinical studies in the SCALE program, in which subcutaneous administration of liraglutide 3 mg per day resulted in a statistically significant weight reduction compared with a placebo. The trial SCALE Obesity and Prediabetes compared the effects of 3 mg of liraglutide vs. a placebo in 3731 overweight or obese people with treated or untreated hypertension and dyslipidemia, without diabetes. In comparison with the placebo group, the drug-treated group had a statistically significant drop in both SBP (−4.2 mmHg vs. −1.5 mmHg, *p* < 0.001) and DBP (−2.6 mmHg vs. −1.9 mmHg, *p* < 0.001) levels [46]. In the SCALE Diabetes trial, liraglutide 3 mg was found to be statistically significantly superior to liraglutide 1.8 mg and to placebo in lowering SBP, but not DBP, in individuals with type II diabetes mellitus who were overweight or obese [47]. The SCALE Maintenance study examined overweight or obese patients with comorbidities other than diabetes mellitus who had lost at least 5% of their body weight through a low-calorie diet before receiving liraglutide 3 mg or a placebo. Again, liraglutide resulted in a statistically significant reduction in SBP values (estimated treatment difference −2.7 mmHg for liraglutide 3 mg vs. placebo, *p* = 0.007), but not in DBP values (estimated treatment difference −0.3 mmHg for liraglutide 3 mg vs. placebo, *p* = 0.64) [48] (Table 1).

##### Semaglutide

Following the STEP clinical trial program in subjects with and without type II diabetes mellitus, subcutaneous semaglutide 2.4 mg once-weekly was approved for the treatment of obesity, as it displayed a clinically meaningful decrease in body weight and improvement in cardiovascular and metabolic risk indicators. In the STEP 1 trial, semaglutide 2.4 mg was administered to overweight or obese non-diabetic participants, and the results showed a significant decrease in SBP (−6.16 mmHg for the semaglutide group vs. −1.06 mmHg for the placebo group, *p* < 0.001) and DBP values (−2.83 mmHg for the semaglutide group vs. −0.42 mmHg for the placebo group, *p* not reported) when compared with the placebo [49]. The STEP 2 study compared the effect of two different doses of semaglutide, 2.4 mg and 1 mg, with a placebo in diabetic patients. Semaglutide at a dose of 2.4 mg appeared to be superior than at the lower dose, as well as the placebo, in promoting weight loss and in lowering SBP values (estimated treatment difference −3.4 mmHg for semaglutide 2.4 mg vs. placebo, *p* = 0.0016) [50]. In the STEP 3 trial, semaglutide 2.4 mg or a placebo were administered to non-diabetic patients, in combination with behavioral therapy. Administration of semaglutide led to a statistically significant weight loss, as well as a drop in SBP (−5.6 mmHg for the semaglutide group vs. −1.6 mmHg for the placebo group, *p* = 0.001) and DBP (−3.0 mmHg for the semaglutide group vs. −0.8 mmHg for the placebo group, *p* = 0.008) values [51]. The goal of the STEP 4 study was to test the maintenance of weight loss after the administration of semaglutide 2.4 mg on a weekly basis in non-diabetic overweight or obese subjects. In this context, semaglutide was administered to all subjects for 20 weeks at a progressively rising dose. Those who reached the 2.4 mg semaglutide dose were randomized to continue at that dose or receive a placebo. Participants who continued taking semaglutide continued to lose weight, and they also experienced a statistically significant reduction in SBP values (estimated treatment difference −3.9 mmHg for semaglutide 2.4 mg vs. placebo, *p* < 0.001), but not DBP values (estimated treatment difference −0.6 mmHg for semaglutide 2.4 mg vs. placebo, *p* = 0.46) [52] (Table 2).

#### 5.4.3. Mechanisms Affecting Blood Pressure

It has been shown that GLP-1 receptors are present in many human organs, and therefore GLP-1 receptor agonists exert both their hypotensive and other beneficial effects through several mechanisms apart from weight loss, many of which are still poorly understood. GLP-1 receptor agonists stimulate the secretion of atrial natriuretic peptide from the cardiac atria, which increases sodium excretion and vasodilation, lowering blood pressure [53]. They also cause vasodilation by their direct action on endothelial and smooth muscle cells, leading to the activation of nitric oxide [53]. The interaction between GLP-1 receptor agonists and the renin–angiotensin–aldosterone system is at least partially responsible for the hypotensive effects of those drugs. Specifically, GLP-1 receptor agonists inhibit the activity of angiotensin II by enhancing its inactivation and opposing its effects on target cells such as cardiomyocytes and glomerular endothelial cells. Moreover, GLP-1 receptor agonists appear to briefly reduce circulating aldosterone levels [44]. Finally, GLP-1 receptor agonists also exert a direct effect on the kidneys and lower blood pressure by enhancing natriuresis, while the effect of these substances on the central nervous system has not yet been confirmed [54].

### 5.5. Tirzepatide

#### 5.5.1. Mechanism of Action

Tirzepatide was first licensed by the FDA and EMA for the treatment of type II diabetes mellitus, and it was granted further permission for the management of obesity in non-diabetic individuals a while ago. Tirzepatide is a novel medication that operates as a dual agonist to the two different types of insulinotropic polypeptide receptors mentioned above, the GLP-1 and the GIP receptors. Tirzepatide increases insulin secretion, inhibits glucagon release, and aids in weight reduction via activating the GLP-1 and GIP receptors [42].

#### 5.5.2. Clinical Evidence on Blood Pressure

Important information on tirzepatide’s effects on blood pressure has been gleaned from a number of significant clinical studies.

##### SURPASS Trials in Diabetic Patients

The primary goal of the SURPASS trials was to evaluate the medication’s effectiveness in helping diabetic patients regulate their blood glucose levels. Secondary outcomes, such as blood pressure changes, were also monitored.

SURPASS-1 was a randomized, double-blind clinical trial that assessed the efficacy of tirzepatide (subcutaneous injections once weekly) in comparison with a placebo in individuals with type II diabetes mellitus not adequately managed with diet and exercise. This study showed that tirzepatide is more effective than the placebo in enhancing glycemic control, reducing body weight, and lowering blood pressure, as the mean SBP drop varied from −4.7 to −5.2 mmHg for the tirzepatide group compared to −2.0 mmHg for the placebo group, while there was no significant difference in DBP between the two groups [55]. In the open-label, phase III SURPASS-2 study, individuals with type II diabetes mellitus were randomly assigned to receive either tirzepatide or semaglutide once a week. Tirzepatide dosage groups of 10 mg and 15 mg showed noticeably higher SBP reductions compared with semaglutide 1 mg [56]. In the SURPASS-3 study, individuals with inadequately managed type II diabetes mellitus were randomized to receive once-weekly tirzepatide or once-daily insulin degludec as an adjuvant to metformin [57]. In the SURPASS-4 study, tirzepatide was compared to insulin glargine in individuals with type II diabetes mellitus and high cardiovascular risk, who were not effectively treated by oral glucose-lowering drugs [58]. Compared with insulin degludec and insulin glargine, all tirzepatide dosages in both trials had favorable effects as regards SBP reduction. In the SURPASS-5 phase III study, subcutaneous tirzepatide was added to titrated insulin glargine, and produced statistically significant improvements in glycemic control among patients with type II diabetes mellitus, as opposed to a placebo. Similarly to the previous studies, this one showcased that all tirzepatide dosages (5, 10, and 15 mg) led to noticeably higher decreases in SBP than the placebo [59] (Table 3).

##### SURMOUNT Trials in Overweight/Obese Individuals

The SURPASS 1–5 trials showed a substantial reduction in weight after tirzepatide treatment; this finding served as the impetus to clinical research on tirzepatide’s efficacy in weight control. SURMOUNT-1 examined ~2500 non-diabetic adults with a BMI of 30 kg/m^2^ or more, or 27 kg/m^2^ or more and at least one comorbidity (cardiovascular disease, arterial hypertension, obstructive sleep apnea, dyslipidemia). Tirzepatide 5 mg, 10 mg, or 15 mg once weekly produced significant and long-lasting dose-dependent reductions in body weight in individuals with a verified diagnosis of obesity when compared to a placebo. Tirzepatide-assisted weight loss in the current trial was associated with statistically greater benefits than a placebo in terms of all assessed cardiovascular risk variables, including SBP and DBP values [60]. The SURMOUNT-1 ABPM Sub-Study examined a selected number of the patients of the aforementioned study, who underwent a 24 h ambulatory blood pressure monitoring. Administration of tirzepatide resulted in a statistically significant reduction in all components of the 24 h blood pressure recording (day, night, and 24 h average blood pressure values) in a manner that was associated with weight loss but not with receiving antihypertensive treatment [61]. The SURMOUNT-2 clinical trial examined both obese and diabetic individuals who were treated with tirzepatide, and showed considerable improvements in glycemic management and weight loss, as well as in blood pressure values, when compared with a placebo [62]. The SURMOUNT-3 study focused on overweight or obese non-diabetic individuals who had achieved a weight loss of ≥5% of their body weight via rigorous lifestyle modification. Tirzepatide considerably lowered body weight in these participants, and this was followed by decreases in blood pressure levels [63]. The first randomized controlled trial evaluating tirzepatide’s effects on cardiovascular outcomes in overweight/obese non-diabetic people was titled SURMOUNT-4. Its findings imply that tirzepatide does not increase the risk of cardiovascular disease, as it presents benefits in a number of indicators of cardiovascular health, such as decreased body weight, decreased blood pressure levels, and improvement of the lipid profile [64] (Table 4).

##### Data from Meta-Analyses

Numerous meta-analyses that examined data from the relevant studies outlined above, as well as a number of smaller ones, have also examined the impact of tirzepatide on blood pressure. Kanbay et al. analyzed data from seven randomized control trials involving 9446 participants. Tirzepatide treatment led to statistically significant reductions in SBP of 4.20 mmHg (95% CI; −5.17 to −3.23) for the 5 mg once-weekly dose, 5.34 mmHg (95% CI; −6.31 to −4.37) for the 10 mg dose, and 5.77 mmHg (95% CI; −6.73 to −4.81) for the 15 mg dose, and in DBP by 2.19 mmHg (95%CI; −2.82 to −1.56) for the 5 mg dose, by 2.53 mmHg (95% CI; −3.16 to −1.90) for the 10 mg dose, and by 2.55 mmHg (95% CI; −3.18 to −1.91) for the 15 mg dose, in comparison with active medications and placebo [65]. Lingvay and colleagues conducted a post hoc analysis of the five SURPASS trials in order to demonstrate the impact of tirzepatide on blood pressure levels and determine if this effect was connected with weight reduction. Tirzepatide caused a higher change in SBP than the other medications used in the trials (tirzepatide 5 mg: −5.1 to −1.3 mmHg, tirzepatide 10 mg: −6.5 to −1.7 mmHg, tirzepatide 15 mg: −11.5 to −3.1 mmHg). The authors claimed that weight reduction was the primary factor mediating changes in SBP, with a modest (r = 0.18–0.22) but significant (*p* < 0.001) association seen between SBP and body weight changes [66]. Lv et al. addressed the effects of tirzepatide in overweight or obese diabetic hypertensive patients in a meta-analysis that reviewed eight studies with 7491 patients. Tirzepatide resulted in a statistically substantial decrease in SBP and DBP levels both when compared with placebo and when compared with GLP-1 receptor agonists [67].

#### 5.5.3. Mechanisms Affecting Blood Pressure

Changes in blood pressure may be caused via a multitude of interrelated routes when tirzepatide is used. First, as mentioned above, it has been shown that reducing body weight decreases blood pressure, and tirzepatide’s potent effects on weight loss most likely have a significant influence in that regard [66]. Second, tirzepatide may have an immediate effect on the cardiovascular system. Notably, tirzepatide primarily affects glucose metabolism, although cardiovascular organs also express GIP and GLP-1 receptors. The activation of these receptors may have an effect on the heart rate, fluid balance, and vascular tone—all of which are crucial for regulating blood pressure [68]. Tirzepatide’s dual action as GLP-1 and GIP receptor agonist can also suppress inflammation by lowering the levels of inflammatory markers such as C-reactive protein, intercellular adhesion molecule 1 (ICAM-1), growth differentiation factor 15, and YKL-40 (also known as chitinase-3-like protein-1). As a result, it can improve the endothelial function and lead to consequent blood pressure reduction. Multiple studies have demonstrated these cardiovascular and anti-inflammatory effects and lent credence to this approach [68,69]. Lastly, tirzepatide may indirectly affect blood pressure through its capacity to improve glycemic control. Research on tirzepatide has shown the existence of benefits in glycemic management, which are associated with decreased insulin resistance and improved indicators of endothelial dysfunction [70]. Moreover, hyperglycemia exacerbates endothelial dysfunction and arterial stiffness, both of which can elevate blood pressure. Tirzepatide may lessen these effects by enhancing glycemic management, resulting in blood pressure reduction [71].

## 6. Bariatric Surgery

### 6.1. Mechanism of Action

Bariatric surgery appears to play an important role in reducing the risk of cardiovascular disease. Such operations are the adjustable gastric band, sleeve gastrectomy, biliopancreatic diversion, duodenal switch, Roux-en-Y gastric bypass, and one-anastomosis gastric bypass [72,73]. These procedures promote sustained weight loss and improve glycemic control, hypertension, and dyslipidemia in individuals with obesity, hypertension, and type II diabetes mellitus, even several years after the surgery. Studies have demonstrated that metabolic and bariatric surgery is more effective compared to non-surgical treatments in managing important cardiovascular risk factors in the short and mid term [73,74].

### 6.2. Clinical Evidence on Blood Pressure

A meta-analysis of 19 randomized clinical trials involving 1353 patients revealed that these procedures can significantly lower SBP and DBP and decrease the need for antihypertensive, antidiabetic, and lipid-lowering medications [74]. Wang et al. highlight that Roux-en-Y gastric bypass has the most significant positive impact on blood pressure, serum lipids, and glucose levels. Furthermore, their findings suggest that people with uncontrolled hypertension and diabetes, mild obesity, aged 45 and over, and smaller waist circumference had a more favorable outcome [74]. Finally, a pooled analysis of 18 non-randomized trials, including 1.5 million patients, indicates that bariatric surgery in individuals with obesity who underwent the procedure is linked to lower long-term all-cause and cardiovascular mortality, and smaller occurrence of obesity-related conditions such as new-onset hypertension, high cholesterol, diabetes, and heart disease when compared with controls [75]. According to the type of bariatric surgery, the operation can lead to a reduction of 1.5–5.1 mmHg in SBP and 2.1–5.6 mmHg in DBP [76].

### 6.3. Mechanisms Affecting Blood Pressure

After undergoing bariatric surgery, individuals with obesity and hypertension observe a decrease in blood pressure as a result of limited calorie intake during recovery. This reduction occurs without any alteration in the mass of visceral adipose tissue. The decrease in leptin levels and the production of reactive oxygen species due to calorie restriction contribute to this effect. Reduced leptin levels lead to a decline in SNS activity, as mentioned above, and subsequently affect blood pressure by diminishing the signals from the central nervous system and carotid body. Moreover, the decrease in reactive oxygen species production enhances vascular endothelial function, further lowering blood pressure. Bariatric surgery speeds up gastrointestinal transit, which increases the release of glucagon-like peptide-1 and peptide YY 3–36 after meals, adding to the blood pressure reduction [73,77].

In the months following bariatric surgery, progressive weight loss is associated with decreased visceral adipose tissue mass, as well as diminished muscle and renal sympathetic nerve activation. This is also linked to reduced activity in the renin–angiotensin–aldosterone system, insulin resistance, augmented natriuretic peptide blood concentration, systemic inflammation, and arterial stiffness, all of which prevent the reappearance of hypertension [72,77]. Changes in gut microbiota resulting from progressive weight loss may also play a part in preventing the recurrence of hypertension [77]. Additionally, it is widely known that obese individuals with obstructive sleep apnea syndrome endure intermittent episodes of hypoxia and/or hypercapnia, triggering the SNS and the renin–angiotensin–aldosterone system, which significantly contribute to hypertension. Improvements in hypertension after bariatric surgery are also attributed to the resolution of obstructive sleep apnea [73,77].

In the context of type II diabetes mellitus, people who lose a similar amount of weight through calorie restriction experience an increase in insulin sensitivity, which mainly contributes to the enhancement of their glucose profile. Additionally, modifications in gut bacteria, gut hormones, and bile acid signaling also play a crucial role in bringing a substantial improvement in glycemic control. Such alterations typically occur shortly after bariatric surgery. Surgery-induced weight loss leads to a noteworthy improvement in the atherogenic lipid profile, characterized by an elevation in the protective high-density lipoprotein and a reduction in triglycerides, total cholesterol, and low-density lipoprotein cholesterol. Furthermore, it has been shown that surgical interventions alleviate inflammatory conditions associated with obesity, which is evident from a decrease in serum inflammatory markers such as C-reactive protein, leptin, and soluble receptor 1 for tumor necrosis factor. Consequently, patients often observe significant enhancements in their quality of life due to the relief of numerous obesity-related health issues [73].

## 7. Discussion

Arterial hypertension and obesity are important cardiovascular risk factors with increasing prevalence and ineffective management. Moreover, these two entities are interconnected through a variety of occasionally intricate pathophysiological mechanisms. Weight loss appears to have a significant impact on improving blood pressure levels through various pathways.

The American and European drug regulatory agencies have approved specific classes of drugs for chronic weight management and treatment of non-genetic obesity, with the goal of both losing weight and maintaining that loss for a significant period of time. Numerous clinical trials and meta-analyses have concluded that the aforementioned authorized medication classes result in a reduction in blood pressure levels, either in a weight loss-dependent manner or as a result of additional pleiotropic effects of the drugs. The combination of naltrexone and bupropion is an exception to this rule, as it seems that this medication either raises or does not influence blood pressure levels despite causing weight reduction. Out of all the drug classes detailed in this review, the GLP-1 receptor agonists liraglutide and semaglutide, as well as tirzepatide, have proven to be efficient in the treatment of type II diabetes mellitus in addition to obesity, with beneficial effects on blood pressure observed in both situations. 

**Figure 4:**
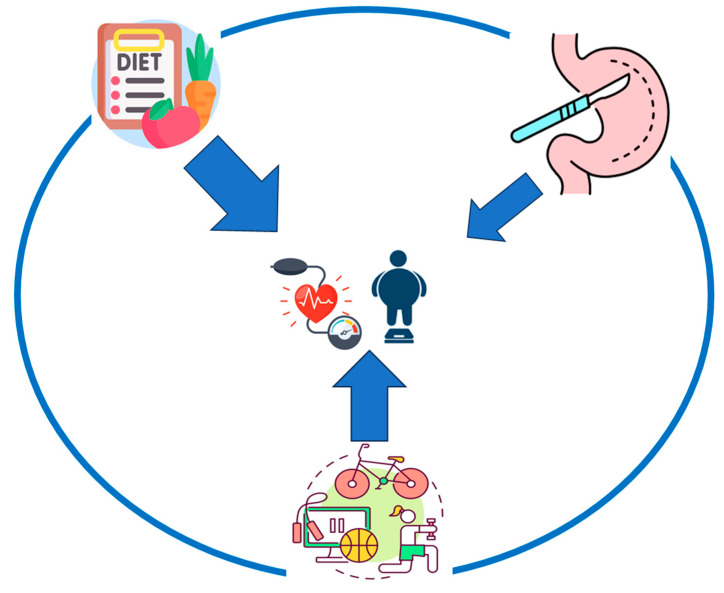
Results of different measures in decrease in blood pressure in obese patients. The image presents the impact of different tools to the decrease in blood pressure. The larger the width of the arrow, the larger the impact. Diet is the tool with the larger impact, followed by physical activity and bariatric surgery.

Apart from pharmaceutical treatment, the basis of obesity treatment must be lifestyle changes by adopting healthy dietary interventions and a physical exercise program. Over and above this, a greater availability of bariatric surgery may contribute to enhancing positive health outcomes for obese individuals, making this field highly compelling from both medical and surgical perspectives. Ultimately, the combination of all the above approaches can lead to a reduction in body weight, the improvement of cardiovascular risk factors such as arterial hypertension, and finally the improvement of the person’s quality of life (Figure 4).

## 8. Limitations

Despite the promising results, there are significant constraints to any inferences regarding blood pressure that may be drawn from the trials showing the benefit of licensed anti-obesity medications for weight reduction. To begin with, these studies were not designed to explore the effect of these drugs on blood pressure, so the relevant conclusions drawn are based mainly on observational data. Furthermore, the study population did not have uniformly defined features as regards the existence of diagnosed arterial hypertension, the use of antihypertensive treatment, and, in the presence of the disease, the adequate control of blood pressure. Based on this evidence, therefore, the above interventions that have been approved for controlling body weight cannot also be used to control blood pressure levels. In addition, the extremely high cost and the locally limited availability of the above pharmaceuticals are factors that negatively affect their widespread use.

## 9. Future Directions

In the treatment of obesity-related hypertension in the future, it is important to implement a more holistic strategy. This would involve personalized lifestyle modification plans, referring patients to specialized dieticians or community/hospital programs that focus on lifestyle education while promoting the use of apps for self-management of blood pressure and weight loss. If these approaches prove ineffective, access to pharmacological weight loss or bariatric surgery should be considered [78]. However, currently available apps have limited functionality, which includes logging blood pressure, giving lifestyle advice, and providing information about hypertension. App development in the future should expand its scope to create a system capable of adapting flexibly to various forms of support, allowing individuals to self-manage their hypertension more effectively [79]. Furthermore, given the importance of obesity treatment in the treatment of arterial hypertension, it is necessary to conduct properly designed clinical studies in the future focusing on the effect of anti-obesity drugs on blood pressure and, above all, on the benefit of these drugs in hypertensive patients. Such studies should be multicenter and randomized, they should study hypertensive patients and how they respond to weight management drugs, and perhaps they may compare anti-obesity therapies with existing antihypertensive medications.

## 10. Conclusions

In conclusion, managing excess body weight will have a major positive impact on regulating a number of cardiometabolic risk factors. The development of novel pharmacological therapies has given new weapons in the quiver of health professionals in this direction. Although the aforementioned pharmacological and surgical weight loss treatments are beneficial for blood pressure levels, they cannot be used instead of or in addition to existing hypertension medications that are intended to treat hypertension. Arterial hypertension is a complex disease caused by multifactorial pathophysiological mechanisms, only a portion of which contribute to the development of obesity. The treatment of hypertension has been established by the guidelines of scientific societies, and has been developed via several properly designed clinical trials aimed at determining the best course of action for hypertensive patients. Future research might lead to the development of combined pharmacological interventions targeted at improving many cardiovascular risk factors at once. Physicians dedicated to management of hypertension in collaboration with cardiometabolic and obesity units could initiate anti-obesity treatments in patients with hypertension and obesity, while using modern technology for self-management. This holistic approach should be offered regardless of the existence of diabetes or prediabetes, to reduce overall risk of cardiovascular disease by modifying two of the most important risk factors (hypertension and obesity).

## Figures and Tables

**Table 1:** Evidence about the impact on blood pressure from clinical trials supporting the approval of liraglutide for the treatment of obesity.

Trial	Total Population, *n*	Hypertension	Anti-Hypertension Therapy	Comparison	Difference in SBP, mmHg	Difference in DBP, mmHg
SCALE Obesity and Prediabetes [46]	3731	mixed	mixed	Liraglutide 3 mg vs. placebo	−4.2 mmHg vs. −1.5 mmHg *p* < 0.001	−2.6 mmHg vs. −1.9 mmHg *p* < 0.001
SCALE Diabetes [47]	846	mixed	mixed	Liraglutide 3 mg vs. Liraglutide 1.8 mg vs. placebo	ETD −2.59 mmHg for liraglutide 3 mg vs. placebo *p* = 0.01	ETD −0.36 mmHg for liraglutide 3 mg vs. placebo *p* = 0.59
SCALE Maintenance [48]	422	mixed	mixed	Liraglutide 3 mg vs. placebo	ETD −2.7 mmHg for liraglutide 3 mg vs. placebo *p* = 0.007	ETD −0.3 mmHg for liraglutide 3 mg vs. placebo *p* = 0.64

DBP, diastolic blood pressure; ETD, estimated treatment difference; mixed, mixed population with or without hypertension, with or without anti-hypertensive therapy; *n*, number of patients; SBP, systolic blood pressure; vs., versus.

**Table 2:** Evidence about the impact on blood pressure from clinical trials supporting the approval of semaglutide for the treatment of obesity.

Trial	Total Population, *n*	Hypertension	Anti-Hypertension Therapy	Comparison	Difference in SBP, mmHg	Difference in DBP, mmHg
STEP 1 [49]	1961	mixed	mixed	Semaglutide 2.4 mg vs placebo	−6.16 mmHg vs. −1.06 mmHg *p* < 0.001	−2.83 mmHg vs. −0.42 mmHg *p* NR
STEP 2 [50]	1210	mixed	mixed	Semaglutide 2.4 mg vs. Semaglutide 1 mg vs. placebo	ETD −3.4 mmHg for semaglutide 2.4 mg vs. placebo *p* = 0.0016	ETD −0.7 mmHg for semaglutide 2.4 mg vs. placebo *p* NR
STEP 3 [51]	611	mixed	mixed	Semaglutide 2.4 mg vs. placebo	−5.6 mmHg vs. −1.6 mmHg *p* = 0.001	−3.0 mmHg vs. −0.8 mmHg *p* = 0.008
STEP 4 [52]	803	mixed	mixed	Semaglutide 2.4 mg vs. placebo	ETD −3.9 mmHg for semaglutide 2.4 mg vs. placebo *p* < 0.001	ETD −0.6 mmHg for semaglutide 2.4 mg vs. placebo *p* = 0.46

DBP, diastolic blood pressure; ETD, estimated treatment difference; mixed, mixed population with or without hypertension, with or without anti-hypertensive therapy; *n*, number of patients; SBP, systolic blood pressure; vs., versus.

**Table 3:** Evidence about the impact on blood pressure from SURPASS trials supporting the approval of tirzepatide for the treatment of type II diabetes mellitus.

Trial	Total Population, *n*	Hypertension	Anti-Hypertension Therapy	Comparison	Difference in SBP, mmHg	Difference in DBP, mmHg
SURPASS-1 [55]	478	mixed	mixed	Tirzepatide 5 mg vs placebo	−4.7 mmHg vs. −2.0 mmHg *p* NR	−2.9 mmHg vs. −1.4 mmHg *p* NR
				Tirzepatide 10 mg vs. placebo	−5.2 mmHg vs. −2.0 mmHg *p* < 0.05	−3.1 mmHg vs. −1.4 mmHg *p* NR
				Tirzepatide 15 mg vs. placebo	−4.7 mmHg vs. −2.0 mmHg *p* NR	−3.4 mmHg vs. −1.4 mmHg *p* NR
SURPASS-2 [56]	1878	mixed	mixed	Tirzepatide 5 mg vs. Semaglutide 1 mg	−4.8 mmHg vs. −3.6 mmHg *p* NR	−1.9 mmHg vs. −1.0 mmHg *p* NR
				Tirzepatide 10 mg vs. Semaglutide 1 mg	−5.3 mmHg vs. −3.6 mmHg *p* < 0.05	−2.5 mmHg vs. −1.0 mmHg *p* NR
				Tirzepatide 15 mg vs. Semaglutide 1 mg	−6.5 mmHg vs. −3.6 mmHg *p* < 0.001	−2.9 mmHg vs. −1.0 mmHg *p* NR
SURPASS-3 [57]	1444	mixed	mixed	Tirzepatide 5 mg vs. Insulin Degludec	−4.9 mmHg vs. +0.5 mmHg	−2.0 mmHg vs. +0.4 mmHg
				Tirzepatide 10 mg vs. Insulin Degludec	−6.6 mmHg vs. +0.5 mmHg	−2.5 mmHg vs. +0.4 mmHg
				Tirzepatide 15 mg vs. Insulin Degludec	−5.5 mmHg vs. +0.5 mmHg	−1.9 mmHg vs. +0.4 mmHg
SURPASS-4 [58]	2002	mixed	mixed	Tirzepatide 5 mg vs. Insulin Glargine	−2.8 mmHg vs. +1.3 mmHg	−1.0 mmHg vs. + 0.7 mmHg
				Tirzepatide 10 mg vs. Insulin Glargine	−3.7 mmHg vs. +1.3 mmHg	−0.8 mmHg vs. +0.7 mmHg
				Tirzepatide 15 mg vs. Insulin Glargine	−4.8 mmHg vs. +1.3 mmHg	−1.0 mmHg vs. +0.7 mmHg
SURPASS-5 [59]	475	mixed	mixed	Tirzepatide 5 mg + Insulin Glargine vs. Placebo + Insulin Glargine	−6.1 mmHg vs. −1.7 mmHg *p* = 0.012	−2.0 mmHg vs. −2.1 mmHg *p* = 0.958
				Tirzepatide 10 mg + Insulin Glargine vs. Placebo + Insulin Glargine	−8.3 mmHg vs. −1.7 mmHg *p* < 0.001	−3.3 mmHg vs. −2.1 mmHg *p* = 0.218
				Tirzepatide 15 mg + Insulin Glargine vs. Placebo + Insulin Glargine	−12.6 mmHg vs. −1.7 mmHg *p* < 0.001	−4.5 mmHg vs. −2.1 mmHg *p* = 0.017

DBP, diastolic blood pressure; mixed, mixed population with or without hypertension, with or without anti-hypertensive therapy; *n*, number of patients; SBP, systolic blood pressure; vs., versus.

**Table 4:** Evidence about the impact on blood pressure from SURMOUNT trials supporting the approval of tirzepatide for the treatment of obesity.

Trial	Total Population, *n*	Hypertension	Anti-Hypertension Therapy	Comparison	Difference in SBP, mmHg	Difference in DBP, mmHg
SURMOUNT-1 [60]	2539	mixed	mixed	Tirzepatide 5 mg or 10 mg or 15 mg vs. placebo	Pooled Tirzepatide groups −7.2 mmHg vs. −1.0 mmHg *p* < 0.001	Pooled Tirzepatide groups −4.8 mmHg vs. −0.8 mmHg *p* < 0.001
SURMOUNT-2 [62]	938	mixed	mixed	Tirzepatide 10 or 15 mg vs. placebo	Pooled Tirzepatide groups −6.3 mmHg vs. −1.2 mmHg *p* < 0.0001	Pooled Tirzepatide groups −2.5 mmHg vs. −0.3 mmHg *p* = 0.0012
SURMOUNT-3 [63]	579	mixed	mixed	Tirzepatide 10 or 15 mg vs. placebo	−5.1 mmHg vs. +4.1 mmHg	−3.2 mmHg vs. +2.3 mmHg
SURMOUNT-4 [64]	783	mixed	mixed	Tirzepatide 10 or 15 mg vs. placebo	−9.7 mmHg vs. −2.0 mmHg *p* < 0.001	−5.5 mmHg vs. −1.7 mmHg *p* < 0.001

DBP, diastolic blood pressure; mixed, mixed population with or without hypertension, with or without anti-hypertensive therapy; *n*, number of patients; SBP, systolic blood pressure; vs., versus.

## References

[1] Obesity. https://www.who.int/health-topics/obesity.

[2] (1998). National Institutes of Health. Clinical Guidelines on the Identification, Evaluation, and Treatment of Overweight and Obesity in Adults—The Evidence Report. Obes. Res..

[3] Powell-Wiley T.M., Poirier P., Burke L.E., Després J.-P., Gordon-Larsen P., Lavie C.J., Lear S.A., Ndumele C.E., Neeland I.J., Sanders P. (2021). Obesity and Cardiovascular Disease: A Scientific Statement from the American Heart Association. Circulation.

[4] Williams B., Mancia G., Spiering W., Agabiti Rosei E., Azizi M., Burnier M., Clement D., Coca A., De Simone G., Dominiczak A. (2018). 2018 Practice Guidelines for the Management of Arterial Hypertension of the European Society of Hypertension and the European Society of Cardiology: ESH/ESC Task Force for the Management of Arterial Hypertension. J. Hypertens..

[5] Olsen M.H., Angell S.Y., Asma S., Boutouyrie P., Burger D., Chirinos J.A., Damasceno A., Delles C., Gimenez-Roqueplo A.-P., Hering D. (2016). A Call to Action and a Lifecourse Strategy to Address the Global Burden of Raised Blood Pressure on Current and Future Generations: The Lancet Commission on Hypertension. Lancet.

[6] Hypertension. https://www.who.int/news-room/fact-sheets/detail/hypertension.

[7] Seravalle G., Grassi G. (2017). Obesity and Hypertension. Pharmacol. Res..

[8] Fantin F., Giani A., Zoico E., Rossi A.P., Mazzali G., Zamboni M. (2019). Weight Loss and Hypertension in Obese Subjects. Nutrients.

[9] Grassi G. (2009). Assessment of Sympathetic Cardiovascular Drive in Human Hypertension: Achievements and Perspectives. Hypertension.

[10] Shariq O.A., McKenzie T.J. (2020). Obesity-Related Hypertension: A Review of Pathophysiology, Management, and the Role of Metabolic Surgery. Gland. Surg..

[11] Reid I.A. (1992). Interactions between ANG II, Sympathetic Nervous System, and Baroreceptor Reflexes in Regulation of Blood Pressure. Am. J. Physiol..

[12] Fisher J.P., Paton J.F.R. (2012). The Sympathetic Nervous System and Blood Pressure in Humans: Implications for Hypertension. J. Hum. Hypertens..

[13] Mancia G., Grassi G. (2014). The Autonomic Nervous System and Hypertension. Circ. Res..

[14] Mancia G., Grassi G. (2013). The Central Sympathetic Nervous System in Hypertension. Handb. Clin. Neurol..

[15] El Meouchy P., Wahoud M., Allam S., Chedid R., Karam W., Karam S. (2022). Hypertension Related to Obesity: Pathogenesis, Characteristics and Factors for Control. Int. J. Mol. Sci..

[16] Da Silva G., Da Silva M., Nascimento D., Lima Silva E., Gouvêa F., De França Lopes L., Araújo A., Ferraz Pereira K., De Queiroz T. (2021). Nitric Oxide as a Central Molecule in Hypertension: Focus on the Vasorelaxant Activity of New Nitric Oxide Donors. Biology.

[17] Stec D.E., Drummond H.A., Vera T. (2008). Role of Carbon Monoxide in Blood Pressure Regulation. Hypertension.

[18] Johnson F.K., Johnson R.A., Durante W., Jackson K.E., Stevenson B.K., Peyton K.J. (2006). Metabolic Syndrome Increases Endogenous Carbon Monoxide Production to Promote Hypertension and Endothelial Dysfunction in Obese Zucker Rats. Am. J. Physiol. Regul. Integr. Comp. Physiol..

[19] Neter J.E., Stam B.E., Kok F.J., Grobbee D.E., Geleijnse J.M. (2003). Influence of Weight Reduction on Blood Pressure: A Meta-Analysis of Randomized Controlled Trials. Hypertension.

[20] Aucott L., Rothnie H., McIntyre L., Thapa M., Waweru C., Gray D. (2009). Long-Term Weight Loss from Lifestyle Intervention Benefits Blood Pressure?: A Systematic Review. Hypertension.

[21] Koskinas K.C., Van Craenenbroeck E.M., Antoniades C., Blüher M., Gorter T.M., Hanssen H., Marx N., McDonagh T.A., Mingrone G., Rosengren A. (2024). Obesity and Cardiovascular Disease: An ESC Clinical Consensus Statement. Eur. Heart J..

[22] McEvoy J.W., McCarthy C.P., Bruno R.M., Brouwers S., Canavan M.D., Ceconi C., Christodorescu R.M., Daskalopoulou S.S., Ferro C.J., Gerdts E. (2024). 2024 ESC Guidelines for the Management of Elevated Blood Pressure and Hypertension. Eur. Heart J..

[23] Viera A.J., Kshirsagar A.V., Hinderliter A.L. (2008). Lifestyle Modifications to Lower or Control High Blood Pressure: Is Advice Associated with Action? The Behavioral Risk Factor Surveillance Survey. J. Clin. Hypertens..

[24] Engeli S., Jordan J. (2013). Novel Metabolic Drugs and Blood Pressure: Implications for the Treatment of Obese Hypertensive Patients?. Curr. Hypertens. Rep..

[25] Allison D.B., Gadde K.M., Garvey W.T., Peterson C.A., Schwiers M.L., Najarian T., Tam P.Y., Troupin B., Day W.W. (2012). Controlled-Release Phentermine/Topiramate in Severely Obese Adults: A Randomized Controlled Trial (EQUIP). Obesity.

[26] Davidson M.H., Tonstad S., Oparil S., Schwiers M., Day W.W., Bowden C.H. (2013). Changes in Cardiovascular Risk Associated with Phentermine and Topiramate Extended-Release in Participants With Comorbidities and a Body Mass Index ≥27 kg/m^2^. Am. J. Cardiol..

[27] Aronne L.J., Wadden T.A., Peterson C., Winslow D., Odeh S., Gadde K.M. (2013). Evaluation of Phentermine and Topiramate versus Phentermine/Topiramate Extended-release in Obese Adults. Obesity.

[28] Garvey W.T., Ryan D.H., Look M., Gadde K.M., Allison D.B., Peterson C.A., Schwiers M., Day W.W., Bowden C.H. (2012). Two-Year Sustained Weight Loss and Metabolic Benefits with Controlled-Release Phentermine/Topiramate in Obese and Overweight Adults (SEQUEL): A Randomized, Placebo-Controlled, Phase 3 Extension Study. Am. J. Clin. Nutr..

[29] Blüher M., Aras M., Aronne L.J., Batterham R.L., Giorgino F., Ji L., Pietiläinen K.H., Schnell O., Tonchevska E., Wilding J.P.H. (2023). New Insights into the Treatment of Obesity. Diabetes Obes. Metab..

[30] Cohen J.B., Gadde K.M. (2019). Weight Loss Medications in the Treatment of Obesity and Hypertension. Curr. Hypertens. Rep..

[31] Sahebkar A., Simental-Mendía L.E., Kovanen P.T., Pedone C., Simental-Mendía M., Cicero A.F.G. (2018). Effects of Orlistat on Blood Pressure: A Systematic Review and Meta-Analysis of 27 Randomized Controlled Clinical Trials. J. Am. Soc. Hypertens..

[32] Sharma A.M., Golay A. (2002). Effect of Orlistat-Induced Weight Loss on Blood Pressure and Heart Rate in Obese Patients with Hypertension. J. Hypertens..

[33] Al-Tahami B.A.M., Ismail A.A.A.-S., Bee Y.T.G., Awang S.A., Salha Wan Abdul Rani W.R., Sanip Z., Rasool A.H.G. (2015). The Effects of Anti-Obesity Intervention with Orlistat and Sibutramine on Microvascular Endothelial Function. Clin. Hemorheol. Microcirc..

[34] Billes S.K., Sinnayah P., Cowley M.A. (2014). Naltrexone/Bupropion for Obesity: An Investigational Combination Pharmacotherapy for Weight Loss. Pharmacol. Res..

[35] Greenway F.L., Fujioka K., Plodkowski R.A., Mudaliar S., Guttadauria M., Erickson J., Kim D.D., Dunayevich E. (2010). Effect of Naltrexone plus Bupropion on Weight Loss in Overweight and Obese Adults (COR-I): A Multicentre, Randomised, Double-Blind, Placebo-Controlled, Phase 3 Trial. Lancet.

[36] Apovian C.M., Aronne L., Rubino D., Still C., Wyatt H., Burns C., Kim D., Dunayevich E., for the COR-II Study Group (2013). A Randomized, Phase 3 Trial of Naltrexone SR/Bupropion SR on Weight and Obesity-related Risk Factors (COR-II). Obesity.

[37] Wadden T.A., Foreyt J.P., Foster G.D., Hill J.O., Klein S., O’Neil P.M., Perri M.G., Pi-Sunyer F.X., Rock C.L., Erickson J.S. (2011). Weight Loss With Naltrexone SR/Bupropion SR Combination Therapy as an Adjunct to Behavior Modification: The COR-BMOD Trial. Obesity.

[38] Hollander P., Gupta A.K., Plodkowski R., Greenway F., Bays H., Burns C., Klassen P., Fujioka K., for the COR-Diabetes Study Group (2013). Effects of Naltrexone Sustained- Release/Bupropion Sustained-Release Combination Therapy on Body Weight and Glycemic Parameters in Overweight and Obese Patients with Type 2 Diabetes. Diabetes Care.

[39] Jiang Q., Velu P., Sohouli M.H., Ziamanesh F., Shojaie S., Fatahi S., Li Q. (2023). The Effects of Bupropion Alone and Combined with Naltrexone on Blood Pressure and CRP Concentration: A Systematic Review and Meta-regression Analysis of Randomized Controlled Trials. Eur. J. Clin. Investig..

[40] Cataldi M., Cignarelli A., Giallauria F., Muscogiuri G., Barrea L., Savastano S., Colao A., on behalf of Obesity Programs of Nutrition, Education, Research and Assessment (OPERA) Group (2020). Cardiovascular Effects of Antiobesity Drugs: Are the New Medicines All the Same?. Int. J. Obes. Suppl..

[41] Arias H.R. (2009). Is the Inhibition of Nicotinic Acetylcholine Receptors by Bupropion Involved in Its Clinical Actions?. Int. J. Biochem. Cell Biol..

[42] Fisman E.Z., Tenenbaum A. (2021). The Dual Glucose-Dependent Insulinotropic Polypeptide (GIP) and Glucagon-like Peptide-1 (GLP-1) Receptor Agonist Tirzepatide: A Novel Cardiometabolic Therapeutic Prospect. Cardiovasc. Diabetol..

[43] Hu E.-H., Tsai M.-L., Lin Y., Chou T.-S., Chen T.-H. (2024). A Review and Meta-Analysis of the Safety and Efficacy of Using Glucagon-like Peptide-1 Receptor Agonists. Medicina.

[44] Puglisi S., Rossini A., Poli R., Dughera F., Pia A., Terzolo M., Reimondo G. (2021). Effects of SGLT2 Inhibitors and GLP-1 Receptor Agonists on Renin-Angiotensin-Aldosterone System. Front. Endocrinol..

[45] Ard J., Fitch A., Fruh S., Herman L. (2021). Weight Loss and Maintenance Related to the Mechanism of Action of Glucagon-Like Peptide 1 Receptor Agonists. Adv. Ther..

[46] Pi-Sunyer X., Astrup A., Fujioka K., Greenway F., Halpern A., Krempf M., Lau D.C.W., Le Roux C.W., Violante Ortiz R., Jensen C.B. (2015). A Randomized, Controlled Trial of 3.0 Mg of Liraglutide in Weight Management. N. Engl. J. Med..

[47] Davies M.J., Bergenstal R., Bode B., Kushner R.F., Lewin A., Skjøth T.V., Andreasen A.H., Jensen C.B., DeFronzo R.A., for the NN8022-1922 Study Group (2015). Efficacy of Liraglutide for Weight Loss Among Patients with Type 2 Diabetes: The SCALE Diabetes Randomized Clinical Trial. JAMA.

[48] Wadden T.A., Hollander P., Klein S., Niswender K., Woo V., Hale P.M., Aronne L., on behalf of the NN8022-1923 Investigators (2013). Weight Maintenance and Additional Weight Loss with Liraglutide after Low-Calorie-Diet-Induced Weight Loss: The SCALE Maintenance Randomized Study. Int. J. Obes..

[49] Wilding J.P.H., Batterham R.L., Calanna S., Davies M., Van Gaal L.F., Lingvay I., McGowan B.M., Rosenstock J., Tran M.T.D., Wadden T.A. (2021). Once-Weekly Semaglutide in Adults with Overweight or Obesity. N. Engl. J. Med..

[50] Davies M., Færch L., Jeppesen O.K., Pakseresht A., Pedersen S.D., Perreault L., Rosenstock J., Shimomura I., Viljoen A., Wadden T.A. (2021). Semaglutide 2·4 Mg Once a Week in Adults with Overweight or Obesity, and Type 2 Diabetes (STEP 2): A Randomised, Double-Blind, Double-Dummy, Placebo-Controlled, Phase 3 Trial. Lancet.

[51] Wadden T.A., Bailey T.S., Billings L.K., Davies M., Frias J.P., Koroleva A., Lingvay I., O’Neil P.M., Rubino D.M., Skovgaard D. (2021). Effect of Subcutaneous Semaglutide vs Placebo as an Adjunct to Intensive Behavioral Therapy on Body Weight in Adults with Overweight or Obesity: The STEP 3 Randomized Clinical Trial. JAMA.

[52] Rubino D., Abrahamsson N., Davies M., Hesse D., Greenway F.L., Jensen C., Lingvay I., Mosenzon O., Rosenstock J., Rubio M.A. (2021). Effect of Continued Weekly Subcutaneous Semaglutide vs Placebo on Weight Loss Maintenance in Adults with Overweight or Obesity: The STEP 4 Randomized Clinical Trial. JAMA.

[53] Kim M., Platt M.J., Shibasaki T., Quaggin S.E., Backx P.H., Seino S., Simpson J.A., Drucker D.J. (2013). GLP-1 Receptor Activation and Epac2 Link Atrial Natriuretic Peptide Secretion to Control of Blood Pressure. Nat. Med..

[54] Goud A., Zhong J., Peters M., Brook R.D., Rajagopalan S. (2016). GLP-1 Agonists and Blood Pressure: A Review of the Evidence. Curr. Hypertens. Rep..

[55] Rosenstock J., Wysham C., Frías J.P., Kaneko S., Lee C.J., Fernández Landó L., Mao H., Cui X., Karanikas C.A., Thieu V.T. (2021). Efficacy and Safety of a Novel Dual GIP and GLP-1 Receptor Agonist Tirzepatide in Patients with Type 2 Diabetes (SURPASS-1): A Double-Blind, Randomised, Phase 3 Trial. Lancet.

[56] Frías J.P., Davies M.J., Rosenstock J., Pérez Manghi F.C., Fernández Landó L., Bergman B.K., Liu B., Cui X., Brown K. (2021). Tirzepatide versus Semaglutide Once Weekly in Patients with Type 2 Diabetes. N. Engl. J. Med..

[57] Ludvik B., Giorgino F., Jódar E., Frias J.P., Fernández Landó L., Brown K., Bray R., Rodríguez Á. (2021). Once-Weekly Tirzepatide versus Once-Daily Insulin Degludec as Add-on to Metformin with or without SGLT2 Inhibitors in Patients with Type 2 Diabetes (SURPASS-3): A Randomised, Open-Label, Parallel-Group, Phase 3 Trial. Lancet.

[58] Del Prato S., Kahn S.E., Pavo I., Weerakkody G.J., Yang Z., Doupis J., Aizenberg D., Wynne A.G., Riesmeyer J.S., Heine R.J. (2021). Tirzepatide versus Insulin Glargine in Type 2 Diabetes and Increased Cardiovascular Risk (SURPASS-4): A Randomised, Open-Label, Parallel-Group, Multicentre, Phase 3 Trial. Lancet.

[59] Dahl D., Onishi Y., Norwood P., Huh R., Bray R., Patel H., Rodríguez Á. (2022). Effect of Subcutaneous Tirzepatide vs Placebo Added to Titrated Insulin Glargine on Glycemic Control in Patients with Type 2 Diabetes: The SURPASS-5 Randomized Clinical Trial. JAMA.

[60] Jastreboff A.M., Aronne L.J., Ahmad N.N., Wharton S., Connery L., Alves B., Kiyosue A., Zhang S., Liu B., Bunck M.C. (2022). Tirzepatide Once Weekly for the Treatment of Obesity. N. Engl. J. Med..

[61] De Lemos J.A., Linetzky B., Le Roux C.W., Laffin L.J., Vongpatanasin W., Fan L., Hemmingway A., Ahmad N.N., Bunck M.C., Stefanski A. (2024). Tirzepatide Reduces 24-Hour Ambulatory Blood Pressure in Adults with Body Mass Index ≥27 Kg/m^2^: SURMOUNT-1 Ambulatory Blood Pressure Monitoring Substudy. Hypertension.

[62] Garvey W.T., Frias J.P., Jastreboff A.M., Le Roux C.W., Sattar N., Aizenberg D., Mao H., Zhang S., Ahmad N.N., Bunck M.C. (2023). Tirzepatide Once Weekly for the Treatment of Obesity in People with Type 2 Diabetes (SURMOUNT-2): A Double-Blind, Randomised, Multicentre, Placebo-Controlled, Phase 3 Trial. Lancet.

[63] Wadden T.A., Chao A.M., Machineni S., Kushner R., Ard J., Srivastava G., Halpern B., Zhang S., Chen J., Bunck M.C. (2023). Tirzepatide after Intensive Lifestyle Intervention in Adults with Overweight or Obesity: The SURMOUNT-3 Phase 3 Trial. Nat. Med..

[64] Aronne L.J., Sattar N., Horn D.B., Bays H.E., Wharton S., Lin W.-Y., Ahmad N.N., Zhang S., Liao R., Bunck M.C. (2024). Continued Treatment With Tirzepatide for Maintenance of Weight Reduction in Adults with Obesity: The SURMOUNT-4 Randomized Clinical Trial. JAMA.

[65] Kanbay M., Copur S., Siriopol D., Yildiz A.B., Gaipov A., Van Raalte D.H., Tuttle K.R. (2023). Effect of Tirzepatide on Blood Pressure and Lipids: A Meta-analysis of Randomized Controlled Trials. Diabetes Obes. Metab..

[66] Lingvay I., Mosenzon O., Brown K., Cui X., O’Neill C., Fernández Landó L., Patel H. (2023). Systolic Blood Pressure Reduction with Tirzepatide in Patients with Type 2 Diabetes: Insights from SURPASS Clinical Program. Cardiovasc. Diabetol..

[67] Lv X., Wang H., Chen C., Zhao Y., Li K., Wang Y., Wang L., Fu S., Liu J. (2024). The Effect of Tirzepatide on Weight, Lipid Metabolism and Blood Pressure in Overweight/Obese Patients with Type 2 Diabetes Mellitus: A Systematic Review and Meta-Analysis. Diabetes Metab. Syndr. Obes..

[68] Taktaz F., Fontanella R.A., Scisciola L., Pesapane A., Basilicata M.G., Ghosh P., Franzese M., Tortorella G., Puocci A., Vietri M.T. (2024). Bridging the Gap between GLP1-Receptor Agonists and Cardiovascular Outcomes: Evidence for the Role of Tirzepatide. Cardiovasc. Diabetol..

[69] Cho Y.K., La Lee Y., Jung C.H. (2023). The Cardiovascular Effect of Tirzepatide: A Glucagon-Like Peptide-1 and Glucose-Dependent Insulinotropic Polypeptide Dual Agonist. J. Lipid Atheroscler..

[70] Pirro V., Roth K.D., Lin Y., Willency J.A., Milligan P.L., Wilson J.M., Ruotolo G., Haupt A., Newgard C.B., Duffin K.L. (2022). Effects of Tirzepatide, a Dual GIP and GLP-1 RA, on Lipid and Metabolite Profiles in Subjects with Type 2 Diabetes. J. Clin. Endocrinol. Metab..

[71] Woodman R.J., Chew G.T., Watts G.F. (2005). Mechanisms, Significance and Treatment of Vascular Dysfunction in Type 2 Diabetes Mellitus: Focus on Lipid-Regulating Therapy. Drugs.

[72] Mancia G., Kreutz R., Brunström M., Burnier M., Grassi G., Januszewicz A., Muiesan M.L., Tsioufis K., Agabiti-Rosei E., Algharably E.A.E. (2023). 2023 ESH Guidelines for the Management of Arterial Hypertension The Task Force for the Management of Arterial Hypertension of the European Society of Hypertension: Endorsed by the International Society of Hypertension (ISH) and the European Renal Association (ERA). J. Hypertens..

[73] Bottino R., Carbone A., Formisano T., D’Elia S., Orlandi M., Sperlongano S., Molinari D., Castaldo P., Palladino A., Barbareschi C. (2023). Cardiovascular Effects of Weight Loss in Obese Patients with Diabetes: Is Bariatric Surgery the Additional Arrow in the Quiver?. Life.

[74] Wang L., Lin M., Yu J., Fan Z., Zhang S., Lin Y., Chen X., Peng F. (2021). The Impact of Bariatric Surgery Versus Non-Surgical Treatment on Blood Pressure: Systematic Review and Meta-Analysis. Obes. Surg..

[75] Wiggins T., Guidozzi N., Welbourn R., Ahmed A.R., Markar S.R. (2020). Association of Bariatric Surgery with All-Cause Mortality and Incidence of Obesity-Related Disease at a Population Level: A Systematic Review and Meta-Analysis. PLoS Med..

[76] Hallersund P., Sjöström L., Olbers T., Lönroth H., Jacobson P., Wallenius V., Näslund I., Carlsson L.M., Fändriks L. (2012). Gastric Bypass Surgery Is Followed by Lowered Blood Pressure and Increased Diuresis—Long Term Results from the Swedish Obese Subjects (SOS) Study. PLoS ONE.

[77] Samson R., Ayinapudi K., Le Jemtel T.H., Oparil S. (2020). Obesity, Hypertension, and Bariatric Surgery. Curr. Hypertens. Rep..

[78] Antza C., Grassi G., Weber T., Persu A., Jordan J., Nilsson P.M., Redon J., Stabouli S., Kreutz R., Kotsis V. (2024). Assessment and Management of Patients with Obesity and Hypertension in European Society of Hypertension Excellence Centres. A Survey from the ESH Working Group on Diabetes and Metabolic Risk Factors. Blood Press..

[79] Hui C.Y., Creamer E., Pinnock H., McKinstry B. (2019). Apps to Support Self-Management for People with Hypertension: Content Analysis. JMIR mHealth uHealth.

